# Quick methylene blue dye elimination via SDS-Ag nanoparticles catalysts

**DOI:** 10.1038/s41598-024-65491-6

**Published:** 2024-07-02

**Authors:** Jamal Q. M. Almarashi, A.-S. Gadallah, Mohamed Shaban, M. A. Ellabban, Kais Hbaieb, Mohamed G. M. Kordy, Mohamed Zayed, Abdel-Aleam H. Mohamed

**Affiliations:** 1 Physics department, College of Science, Taibah University, 30001 Madina, Saudi Arabia; 2 Department of Laser Sciences and Interactions, National Institute of Laser Enhanced Sciences, Cairo University, Giza, 12613 Egypt; 3 Department of Physics, Faculty of Science, Islamic University of Madinah, 42351 Madinah, Saudi Arabia; 4 Nanophotonics and Applications (NPA) Lab, Physics Department, Faculty of Science, Beni-Suef University, Beni-Suef, 62514 Egypt; 5 Physics Department, Faculty of Science, Tanta University, Tanta, 31527 Egypt; 6 Mechanical department, College of Engineering, Taibah University, P.O. Box 344, Al-Madinah Al-Munawwara, Kingdom of Saudi Arabia; 7 Biochemistry Department, Faculty of Science, Beni-Suef University, P.O. Box 62521, Beni-Suef, Egypt; 8 Physics Department, Faculty of Science, Beni-Suef University, Beni-Suef, 62511 Egypt

**Keywords:** Pulsed laser, Ag nanoparticles, Optical properties, Catalytic dye removal, Methylene blue, Materials science, Materials for energy and catalysis

## Abstract

Methylene blue dye, being toxic, carcinogenic and non-biodegradable, poses a serious threat for human health and environmental safety. The effective and time-saving removal of such industrial dye necessitates the use of innovative technologies such as silver nanoparticle-based catalysis. Utilizing a pulsed Nd:YAG laser operating at the second harmonic generation of 532 nm with 2.6 J energy per pulse and 10 ns pulse duration, Ag nanoparticles were synthesized via an eco-friendly method with sodium dodecyl sulphate (SDS) as a capping agent. Different exposure times (15, 30, and 45 min) resulted in varying nanoparticle sizes. Characterization was achieved through UV–Vis absorption spectroscopy, scanning electron microscopy (SEM) imaging, and energy dispersive X-ray (EDX). Lorentzian fitting was used to model nanoparticle size, aligning well with SEM results. Mie’s theory was applied to evaluate the absorption, scattering, and extinction cross-sectional area spectra. EDX revealed increasing Ag and carbon content with exposure time. The SDS-caped AgNPs nanoparticles were tested as catalyst for methylene blue degradation, achieving up to 92.5% removal in just 12 min with a rate constant of 0.2626 min^−1^, suggesting efficient and time-saving catalyst compared to previously reported Ag-based nanocatalysts.

## Introduction

The development of structures at the nanoscale is a critical advancement in technology, enabling the creation of smaller, cost-effective, energy-efficient products with superior capabilities and reduced raw material usage compared to traditional bulk material-based products. Nanoparticles (NPs), due to their high surface area to volume ratios, exhibit enhancement in surface/contact area influenced properties, such as those related to plasmon resonance and catalytic applications^1,2^. Their small size, comparable to cells and viruses, coupled with their large surface area, makes them suitable for various applications in environmental and life sciences. Consequently, nanotechnology-based devices and systems are instrumental in addressing major challenges in diverse fields such as medicine, industry, food technology, communications, and environmental pollution.

Numerous chemical, physical, and biological techniques are employed for nanoparticle synthesis^3^. The most common chemical method involves chemical reduction using organic and inorganic reducing agents in aqueous or non-aqueous solutions. However, there is an increasing demand for eco-friendly methods that avoid the use of toxic chemicals in synthesis protocols. These green methods include biological approaches using organisms to produce nanoparticles, ranging from simple prokaryotic bacterial cells to eukaryotic fungi and plants^4–13^. Additionally, irradiation in liquids for nanoparticle production is an eco-friendly method. Laser irradiation in liquids, categorized into laser ablation of metals in solution, laser fragmentation when a powder suspension is used instead of a solid foil as the ablating target, and laser melting in an aqueous solution, is a relatively simple technique producing controllable particle size^14^. The properties of the nanoparticles produced by laser irradiation are primarily controlled by the laser’s properties, exposure time, and the effective liquid medium. Laser irradiation in liquids is a quick and straightforward technique for producing homogeneous nanomaterials with reduced formation of side products. It allows rigorous size control of the nanomaterials over a wide range from micrometers to nanometers^15^.

Irradiating an aqueous solution with a pulsed laser ionizes water and liberates electrons that compensate Ag^+^ ions of the metal salt dissolved in water, generating Ag nanoparticles (AgNPs). With increasing laser power density, the size of the AgNPs increases. If the irradiation time increases, then the rates of water ionization and electron liberation from ionized water increase, which in turn increases the rate of compensation between liberated electrons and the silver positive ions, thereby increasing the size of AgNPs^16,17^.

AgNPs are among the most widely used nanomaterials due to their high electrical conductivity, antibacterial properties, and distinctive optical properties required in many applications^18,19^. It is believed that the antibacterial property of silver arises from its high affinity toward phosphorus or sulfur, which are abundant in cell membranes. AgNPs react with proteins outside or inside the cell membrane that contain sulfur or interact with DNA phosphorus moieties. Therefore, AgNPs can affect cell viability and inhibit enzyme functions and DNA replication^19,20^. AgNPs are also incorporated into materials for wound dressing, textile, and polymer networks^21^. In addition, AgNPs are known for their unique surface plasmon resonance, which amplifies their optical absorption or strong scattering, giving them unique optical properties. These characteristics have significant potential applications in medical diagnostics, chemical and biochemical sensing, and biological imaging^22^. AgNPs are also recognized for their ease of synthesis and processing, high thermal and chemical stability, and swift kinetic reaction rate^23^.

Importantly, AgNPs demonstrate considerable photocatalytic activity. When incorporated into a less active support with well distribution, toxic agents can be targeted for removal and degradation^24^. AgNPs supported catalysts stand out to be efficient, cost-effective, and environmentally friendly photocatalysts for eliminating toxic organic materials from the ecosystem. The technology involving such catalysts has several advantages over traditional techniques, including the complete degradation of pollutants. It can also be used for H_2_ synthesis by reducing water and CO_2_, and in purifying air and water^25^.

In the photocatalysis process, valence electrons are excited by light into the conduction band. These electrons reduce the surface-adsorbed species, while the holes in the valence band act as oxidizing agents^26^. The most common and effective nano-photocatalysts include ZnO, TiO_2_, and CeO_2_ nanoparticles^27^. However, when loading the AgNPs onto these oxides, e.g. Ag/CdSe and Ag/TiO, the rate of photocatalysis is significantly increased compared to the stand-alone oxides, the absorption in the visible spectral range is boosted, the electron–hole recombination is suppressed, and the photoactivity is enhanced^28^.

The elimination of contaminants such as dyes, hydrocarbons, pathogens, and pesticides using NPs is of great interest. Methylene blue (MB), a basic/cationic dye that has a diethyl amine group and an azo (N=N), is hazardous to livelihood and needs to be destroyed or at least reduced from nature^29^. It is generally used as a dye material for wood, silk, and cotton. Excessive levels of exposure to MB can cause diseases such as digestive and nausea, vomiting, and profuse sweating^30–32^.

Various methods are used to destroy and remove MB, including chemical methods, physicochemical methods, and biological methods^33–37^. Atta et al.^38^ investigated the degradation of methylene blue using silver and magnetite nanoparticles functionalized with a protic poly (ionic liquid) based on a quaternized diethyl ethanolamine cation combined with 2-crylamido-2-methylpropane sulfonate-co-vinylpyrrolidone (QAMPSA/VP) as a capping and a reducing agent. Although complete MB degradation is achieved, the preparation method for this catalytic material is cumbersome. Vanaja et al.^39^ have explored the degradation of methylene blue using silver nanoparticles synthesized biologically by using *Morinda tinctoria* leaf extract under different pHs. It needed a time of 72 h to degrade the dye to nearly 95%^39^. According to Al-Zaban et al.^40^, silver nanoparticles synthesized by honey were used as environmentally reducing and stabilizing agents. In their presence, MB has been degraded by 92% after 72 h.

The urgency to develop innovative, efficient, rapid, and cost-effective AgNP-based catalysts for the eradication of organic pollutants from water solutions is of paramount importance. The aim of this research is surpassing the performance of existing catalysts, pushing technology across new frontiers towards environmental preservation. Here, SDS-capped AgNPs samples were synthesized using a 532 nm nanosecond pulsed laser, in a solution of silver acetate and sodium dodecyl sulfate (SDS). The SDS-capped AgNPs were characterized using SEM, EDX, and UV/Vis spectroscopy, and their sizes were determined using Lorentzian fitting of the experimental absorption data. Mie theory was used to evaluate the absorption, scattering, and extinction cross-sectional areas for the AgNPs of different sizes. The AgNPs, prepared over varying operating times from 15 to 45 min, were used to catalyze the degradation of methylene blue dye. The degradation efficiencies and rates were estimated and compared with previously reported Ag-based catalysts, highlighting the significance of the optimized SDS-capped AgNPs sample in increasing the degradation rate and reducing the degradation time of MB dye. These nanoparticles exhibit remarkable catalytic performance, achieving 92.5% MB dye removal in 12 min, which, to the best of our knowledge, matched the highest reported rate for AgNPs-based catalysts. Our method potentially prevails over the other methods for being simple and scalable. Industries such as textile and water treatment could benefit from this efficient and environmentally friendly solution. Future research can explore broader applications and optimization of this method.

## Experimental details

### Materials

The precursor of silver that has been used is silver acetate (CH_3_CO_2_Ag), with purity of ≥ 98%, and molecular weight of 166.912 g/mol. provided by BHD laboratory, England. The stabilizing agent that has been used to cap Ag nanoparticles is sodium dodecyl sulfate, SDS, (NaC_12_H_25_SO_4_), with purity of ≥ 98.5%, and molecular weight of 288.38 g/mol. It was supplied by Sigma–Aldrich, China. Methylene blue (MB) dye was purchased from Al-Nasr Company (Giza, Egypt) and sodium borohydride (NaBH_4_) was purchased from Research Lab Fine Chem Industries (Mumbai, India). In all of the tests, double-distilled water was utilized.

### Synthesis of laser-prepared SDS-capped AgNPs samples

A quantity of 1 × 10^−4^ M from CH_3_CO_2_Ag and 1 × 10^−4^ M of SDS were dissolved in 100 mL of distilled water using magnetic stirring for about 30 min at room temperature. The produced solution was then exposed to a laser beam for 45 min (sample A02), 30 min (sample B02), and 15 min (sample C02), Table 1. The used Nd:YAG laser operates at the second harmonic generation of 532 nm, with energy per pulse of 2.6 J and a repetition rate of 10 Hz. A volume of 5 mL of the solution in the cuvette was exposed to the laser beam.

**Table 1:** Precursors concentrations and preparation conditions of the samples.

Sample name	Precursor concentration 10^−4^ (M)		Laser parameters		
	Silver acetate	SDS	Exposure time (min)	Frequency (Hz)	Energy per pulse (J)
A02	1	1	45	10	2.6
B02	1	1	30	10	2.6
C02	1	1	15	10	2.6

### Characterization of laser-prepared SDS-capped AgNPs samples

For absorbance measurements, a double beam spectrophotometer, model Shimadzu 3150, with a scanning step of 1 nm in the range from 300 to 700 nm has been used. The surface morphologies of synthesized nanocomposites were analyzed by scanning electron microscopy (SEM) that was conducted on SEM-Sigma 500 VP (Carl ZEISS, Baden-Württemberg, Germany). The chemical or elemental composition of the samples was investigated using a high-energy dispersive X-ray detector integrated into the SEM device (EDX-SEM, AMETEK, Inc., PA, USA). Characterization of Ag NPs using X-ray diffraction (XRD) with Cu-Kα radiation, operated at 40 kV and 40 mA, and a scanning rate of 0.01°/s in the 2θ-range from 5 to 80°, model Philips X’Pert Pro MRD, Malvern, UK. Additionally, dynamic light scattering (DLS) via the Zeta Sizer Nano-series (Malvern, Nano-ZS90, UK) determined the particle size distribution and zeta potential of the Ag NPs. To identify functional groups, Fourier transform infrared (FTIR) spectra were measured using a Bruker Vertex 70 instrument (Bruker, Leipzig, Germany).

### Evaluation of catalytic efficiency of laser-prepared SDS-capped AgNPs samples

The laser-produced SDS-coated AgNPs were utilized as catalysts in the reduction process of 2 mL of 100 ppm MB dye by 1 mL of 0.1 M NaBH_4_, conducted in a 3.5 mL cuvette cell at a controlled temperature of 25 ± 2 ℃.

A volume of 20 µL of the previously mentioned catalysts was used in these catalytic reactions. To aid in data analysis, a control test was performed where distilled water was used in place of the catalyst, thereby eliminating any catalytic activity. Absorbance spectra were captured using a UV/visible/IR spectrophotometer (Perkin Elmer, lambda 950, USA), within a wavelength spectrum of 500–750 nm. To avoid any unintended photocatalytic reactions, the solutions, which consisted of MB and the laser-produced SDS-capped Ag NPs, were stored in dark conditions.

## Results and discussion

Upon the dissolution of silver acetate and SDS in water at the specified concentration, the solution contains positive silver ions. When this solution is subjected to a laser beam with a high energy level of 260 mJ and a short pulse duration of 10 ns, ionization of the water molecules takes place. This ionization process generates negative electrons. These electrons are subsequently acquired by the positive silver ions, thereby neutralizing them. This process is essentially a reduction of the positive silver ions through electron gain, resulting in the production of neutral silver nanoparticles.

### The absorption spectra

**Figure 1:**
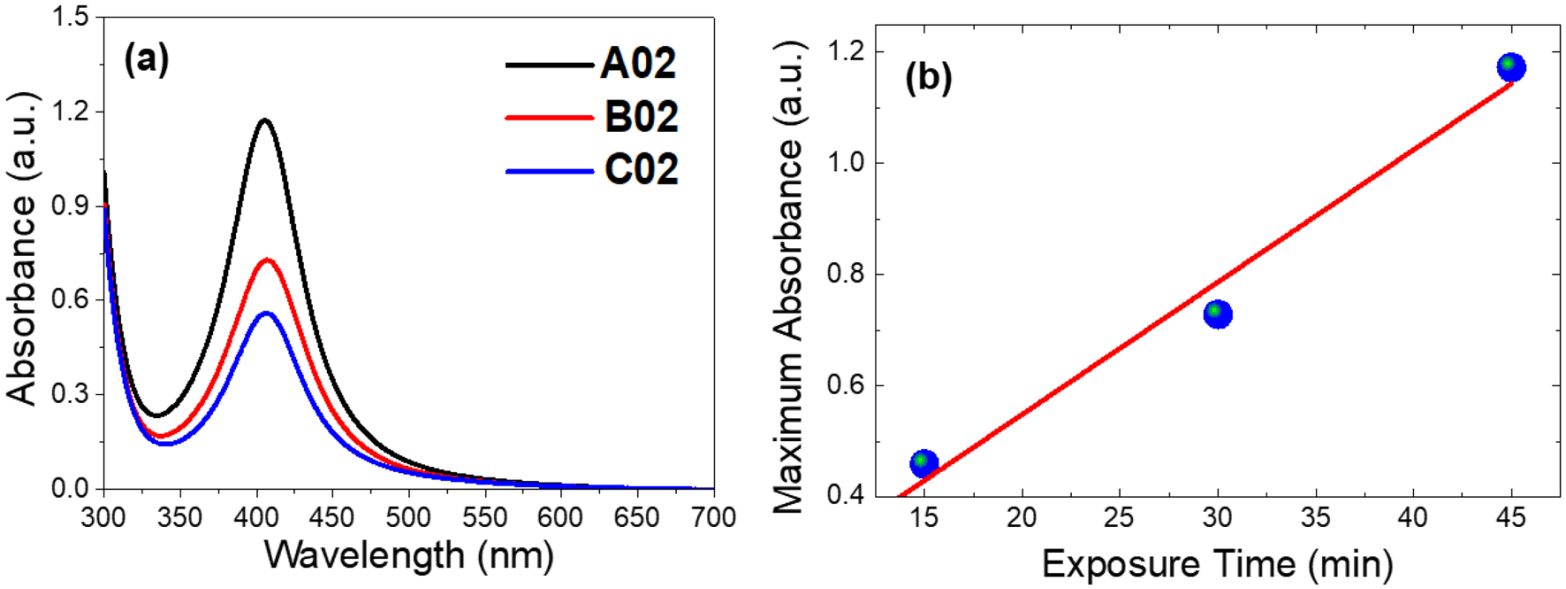
(**a**) Absorbance spectra and (**b**) maximum absorbance versus exposure time of SPR modes of Ag NPs for the samples A02, B02, and C02. Circles represent experimental data, whereas the solid line presents linear fitting.

Figure 1 shows the optical absorbance spectra of the investigated samples. Note that the absorbance is defined as A = Log (I_0_/I_T_), where A is the absorbance, I_0_ is the incident intensity and I_T_ is the transmitted intensity, as reported in^41–43^. The peak of the three samples occurs at 406 nm that is attributed to surface plasmon resonance of free electrons of Ag metal in the conduction band near the surface of Ag nanoparticles. The absorbance increases with the increase of the exposure time. Sample A02 with an exposure time of 45 min has an absorbance peak of 1.173, while sample B02 with exposure time of 30 min has a peak absorbance of 0.7286. The sample C02 with exposure time of 15 min has an absorbance peak of 0.4591. Figure 1b shows the absorbance peak versus the exposure time. For the silver nanoparticle size calculation in terms of the peak position in the absorbance measurement, the following equation has been used:

1 $$D = \frac{{\lambda_{SPR} - 382.6}}{1.18},$$

where D is the modeled effective diameter of silver nanoparticle in nanometer, and λ_SPR_ is the surface plasmon resonance peak wavelength^44^.

The surface plasmon resonance peak wavelength for the three samples occurs at 406 nm. Hence, the calculated particle diameter is 19.8 nm for the three samples. It is noteworthy to mention that the estimated particle size according to Eq. (1) depends only on the peak position in the absorption measurement and does not depend on the full width at half maximum (FWHM) intensity. Experimentally different particle sizes can occur at the same peak absorption wavelength and thus Eq. (1) cannot distinguish between different particle sizes that have the same peak absorption wavelength. For more accurate particle size estimation, another approach has to be used to distinguish between different particle sizes that have the same absorption peak. Mie theory is one of these approaches in which the particle size estimation does not only depend on the peak position, but also depends on the FWHM. It is known that there are two types of broadening, namely, homogeneous broadening and inhomogeneous broadening. When the active species are in a liquid or in a crystal, homogeneous broadening arises and Lorentz function fits well with the absorbing spectra. On the other hand, when the active species are in amorphous solid, inhomogeneous broadening arises and Gauss function fits well with the absorption curve. In the present study, the type of broadening is homogeneous and Lorentz function will be used for fitting the absorption.

**Figure 2:**
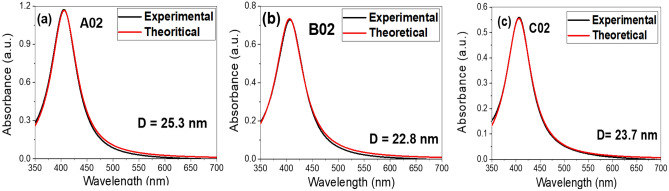
Experimental and theoretical absorbance of the samples (**a**) A02, (**b**) B02, and (**c**) C02.

Lorentz function has the form:

2 $$A\left( {\lambda ,\gamma } \right) = \frac{2}{\pi }a\frac{\gamma }{{\left[ {4\left( {\lambda - \lambda_{SPR} } \right)^{2} + \gamma^{2} } \right]}},$$

where $A\left(\lambda ,\gamma \right)$ is the absorbance as a function of the wavelength *λ* and the FWHM *γ* and $a$ is the area under the absorption curve. The area under absorption curve depends on the number of nanoparticle species that absorb light whereas the FWHM *γ* depends on the lattice defects, impurities, and the scattering between electrons and phonon or between electrons*.* Figure 2 shows fitting of the experimental data using Eq. (2). It appears that there is a good matching between the experimental results and the estimated values from Eq. (2). Using Mie theory, the FWHM, γ, is related to the particle modeled effective diameter D through the following equation:

3 $$\gamma = \gamma_{0} + \frac{{2bv_{F} }}{D},$$

where $\text{b}$ is a constant equal to 0.75 for silver^45^, v_F_ is the Fermi velocity = 1.39 × 10^6^ m/s^46^, and γ_0_ = $\frac{{\text{V}}_{\text{f}}}{{\text{L}}_{\infty }}$ . ${L}_{\infty }$ is the mean free path of the electron in a bulk material and is equal to 52 nm for silver metal^45,46^. Hence, γ_0_ = 2.67 × 10^13^ s^−1^. Table 2 shows the modeled effective particle diameter calculated from Eq. (3) using the parameters of fitting the experimental absorption to Eq. (2). The values of the particle sizes of sample A02, B02, and C02 are 25.3 nm, 22.8 nm, 23.7 respectively.

**Table 2:** SPR wavelength and calculated particle diameter for the samples using Eq. 3.

Sample name	${\lambda }_{SPR}$ (nm)	$\gamma$ (nm)	$\gamma$ (s^−1^) × 10^14^	a	D (nm)
A02	406	60	1.10	110	25.3
B02	406	65	1.18	75	22.8
C02	406	63	1.14	55	23.7

For more theoretical analysis, Mie theory has been used and compared to experimental absorbance. There are three optical cross-sectional areas, which are the absorption, scattering, and extinction cross sectional area that is the sum of both absorption and scattering cross sectional area.

These cross-sectional areas are given by the following equation:

4 $$\sigma_{a} = 3\pi^{2} \varepsilon_{m}^{\frac{3}{2}} \frac{{\varepsilon_{2} }}{{\left( {\varepsilon_{1} + 2\varepsilon_{m} } \right)^{2} + \varepsilon_{2}^{2} }}\frac{{D^{3} }}{\lambda }$$

5 $$\sigma_{s} = \frac{2}{3}\pi^{5} \varepsilon_{m}^{2} \frac{{\left( {\varepsilon_{1} - \varepsilon_{m} } \right)^{2} + \varepsilon_{2}^{2} }}{{\left( {\varepsilon_{1} + 2\varepsilon_{m} } \right)^{2} + \varepsilon_{2}^{2} }}\frac{{D^{6} }}{{\lambda^{4} }}$$

6 $$\sigma_{e} = \sigma_{a} + \sigma_{s}$$

where ${\sigma }_{a}$, ${\sigma }_{s}$ , ${\sigma }_{e}$ are absorption, scattering and extinction cross sectional area respectively, and ${\varepsilon }_{1}$, ${\varepsilon }_{2}$ , ${\varepsilon }_{m}$ are the real, imaginary, and surrounding medium real dielectric constant, respectively, whereas $D$ is the modeled effective particle diameter and λ is the incident wavelength^47^.

**Figure 3:**
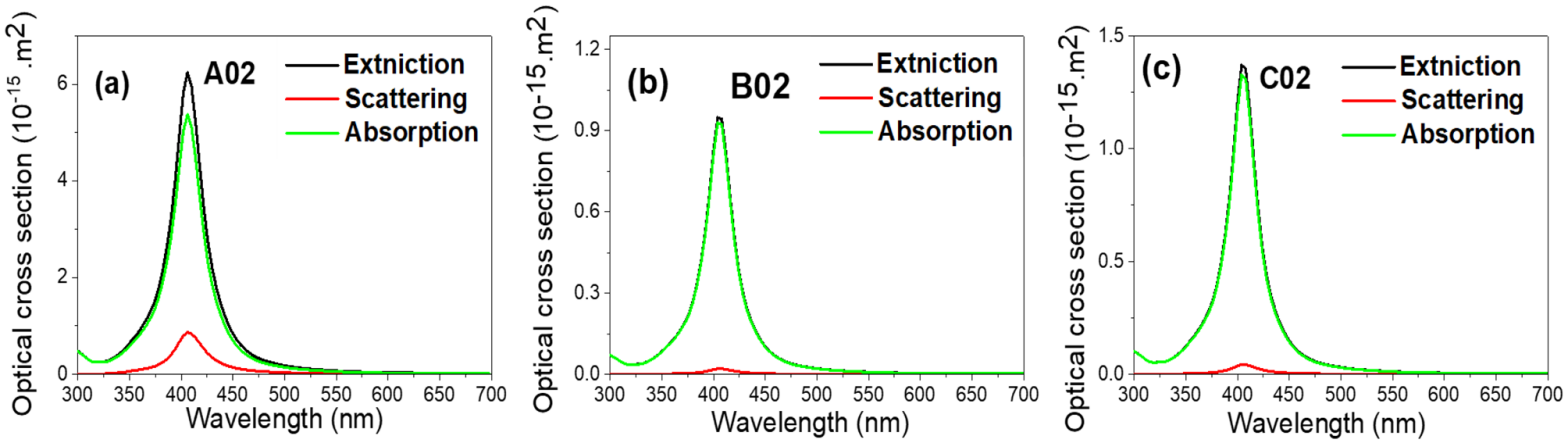
Optical cross-sections of samples (**a**) A02, (**b**) B02, and (**c**) C02.

Figure 3 shows these cross-sectional areas for the three samples. The values of real and imaginary dielectric constants as a function of the wavelength are extracted from the work of Jonhnson and Christy^48^. The value of the surrounding real dielectric constant of the medium (water) as a function of wavelength is that used by the work of Meissner and Wentz^49^. The ratio of the absorption cross-sectional area to the scattering cross sectional area is 5.37 × 10^−15^/8.7 × 10^−16^ = 6.17 for sample A02, Fig. 3a, 9.28 × 10^−16^/2.06 × 10^−17^ = 45.05 for sample B02, Fig. 3b, and 1.3 × 10^−15^/4.9 × 10^−17^ = 26.53 for sample C02, Fig. 3c. Hence the smaller the diameter of the particle is, the larger is the ratio of the absorption to the scattering cross-sectional area. Moreover, both the absorption and scattering cross sectional area increase with increasing particle diameter.

### SEM images

**Figure 4:**
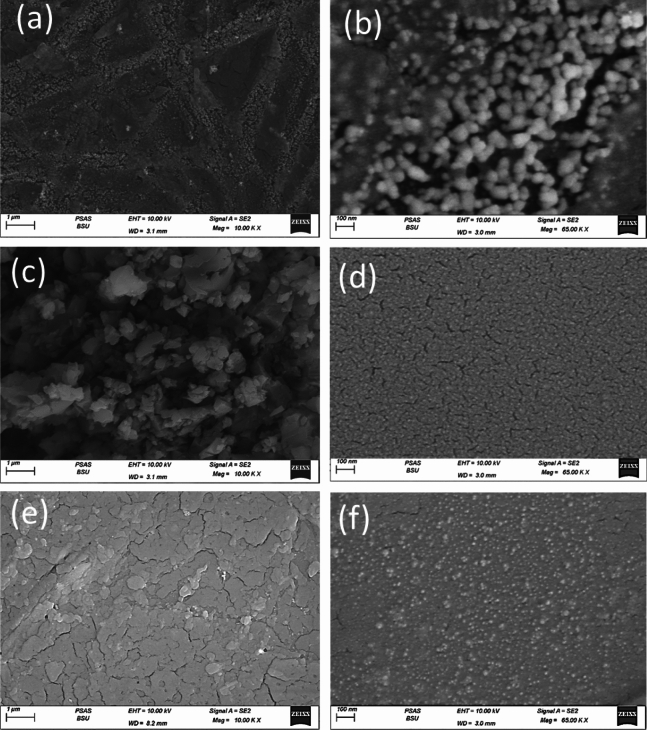
SEM images of samples at different magnifications for samples (**a**,**b**) A02, (**c**,**d**) B02, and (**e**,**f**) C02.

**Figure 5:**
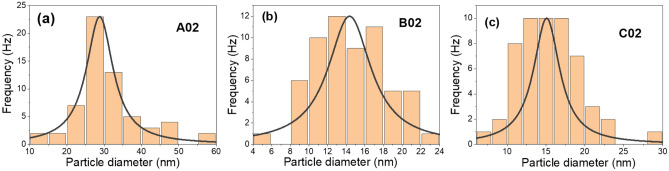
Particle distribution of the samples (**a**) A02, (**b**) B02, and (**c**) C02.

SEM images of the prepared samples are displayed in Fig. 4. Two magnifications, 10 k and 65 k, are presented. The lower magnification provides an overview of the layer deposited on the glass substrate, while the higher magnification offers detailed information about the particles that constitute the layer. For sample A02, as shown in Fig. 4a at lower magnification, branched microfibers with small particles embedded within them are observed^17^. As depicted in Fig. 4b, a distribution of Ag nanoparticle sizes is evident, ranging from a minimum size of approximately 10 nm to a maximum size of about 60 nm. The most frequently occurring average size is around 30 nm. Figure 5a illustrates this nanoparticle distribution using ImageJ software, with the peak of the distribution occurring at a size of 28.8 nm. Additionally, the full width at half maximum (FWHM) of the distribution is approximately 10 nm.

SEM images for sample B02 are presented in Fig. 4c, d. The lower magnification reveals particles deposited on the substrate, forming a flower-like structure. The higher magnification displays the particles that constitute this structure, with sizes ranging from a minimum of 4 nm to a maximum of 24 nm, and an average particle size of about 14.7 nm. Figure 5b depicts the particle size distribution of sample B02, with the most frequently occurring average particle size at 14.5 nm. The FWHM for this distribution is about 5 nm, indicating a better particle uniformity for sample B02 compared to sample A02.

In sample C02, as depicted in Fig. 4e, the deposited layer forms a continuous film with random grain sizes. In Fig. 4f, small particles with certain distributions are observed. Figure 5c presents this nanoparticle diameter distribution for sample C02. The distribution reveals smaller particles of about 7 nm up to larger particles of about 29 nm, and the peak of the distribution occurs at around 15 nm. The FWHM of this distribution is 4 nm, suggesting a better uniformity than that of samples A02 and B02. This condition corresponds to shorter exposure time to laser beam compared to the other two samples.

### EDX analysis

**Figure 6:**
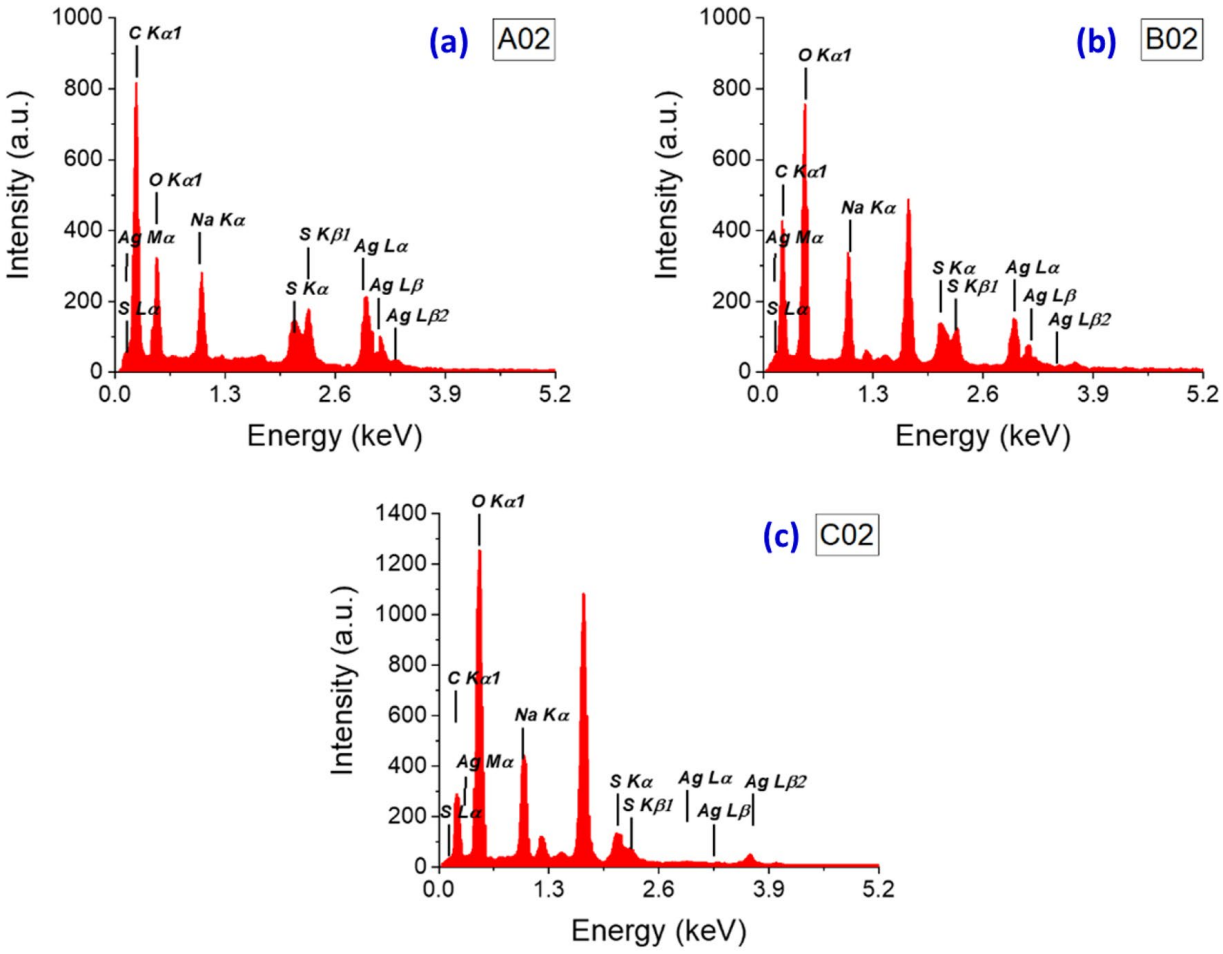
EDX spectra of samples (**a**) A02, (**b**) B02, and (**c**) C02.

Electron dispersive X-ray spectroscopy (EDX) has been employed for both qualitative and quantitative analyses. This technique has enabled the identification of several elements, including Ag, Na, S, and Carbon. The EDX spectra for the samples are depicted in Fig. 6. A peak, indicative of Ag, is observed at 3 keV^35^, and this is consistent across all samples. Other elements, specifically Na, O, C, and S, are also evident in the spectra. These elements are attributed to SDS. For a comprehensive quantitative analysis, Table 3 provides both the atomic and weight percentages of these elements. The table clearly shows that the Ag content in sample A02 is higher than that in B02 and C02. The weight percentages of Ag/Na are 1.378, 0.776, and 0.048 for A02, B02, and C02, respectively. In essence, a decrease in the exposure time of the samples to the laser beam leads to a reduction in the Ag/Na ratio, suggesting an increase in SDS capping. This observation is consistent with the findings from the SEM images and is corroborated by the UV–visible absorption spectra shown in Fig. 1. The C content is also highest in sample A02 compared to B02 and C02. Conversely, O and Na have the lowest content in sample A02 compared to B02 and C02.

**Table 3:** Quantitative elemental analysis using EDX for the samples.

Sample name	Element	Weight %	Atomic %
A02	C	46.96	63.51
	O	24.16	24.53
	Na	9.21	6.51
	S	6.98	3.54
	Ag	12.69	1.91
B02	C	27.69	39.38
	O	43.24	46.15
	Na	12.72	9.45
	S	6.48	3.45
	Ag	9.87	1.56
C02	C	22.52	30.45
	O	52.89	53.7
	Na	18.57	13.12
	S	5.12	2.59
	Ag	0.89	0.13

### DLS and zeta potential

**Figure 7:**
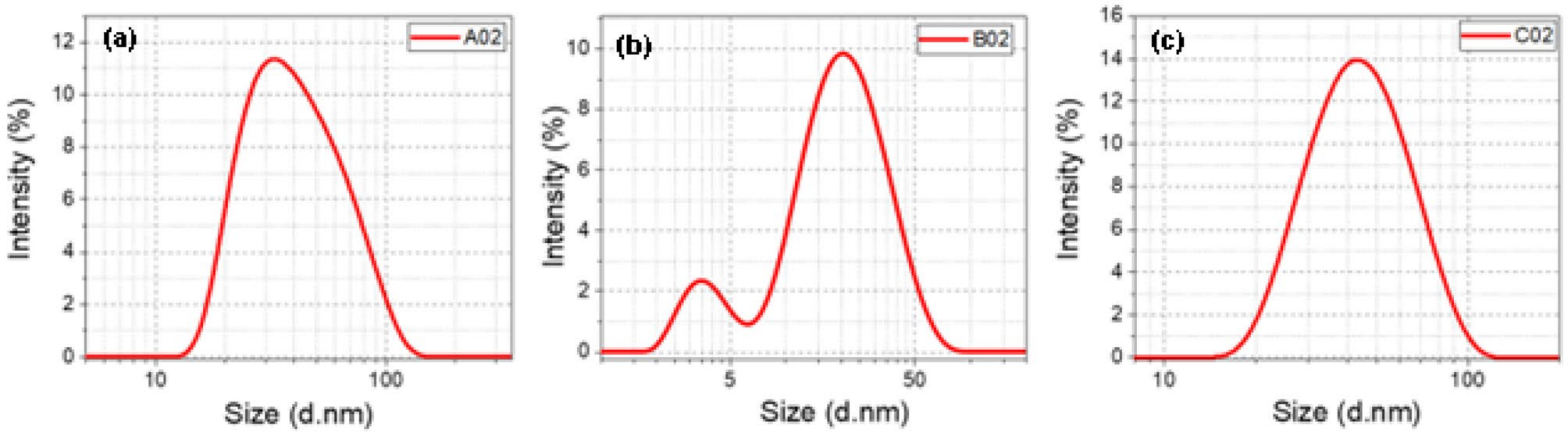
Results of DLS of samples (**a**) A02, (**b**) B02, and (**c**) C02 for particle distribution analysis.

Figure 7 (left) shows dynamic light scattering (DLS) of sample A02. The first peak occurs at 32.7 nm. Using SEM images described in Sect “SEM images”, the most frequently occurring average size is around 30 nm. As shown in Fig. 5a for sample A02, the peak of the particle distribution occurs at 28.8 nm, which is close to the results of this DLS measurement. Figure 7b shows DLS of sample B02. The dominant peak occurs at the vicinity of 20 nm. The most frequently occurring average size using SEM images described in Sect “SEM images” is in the vicinity of 15 nm, which is close to the result of DLS measurements. Unlike the other two figures, Fig. 7b exhibited a secondary lower intensity peak at 3.4 nm. Figure 7c shows DLS of sample C02. The dominant peak occurs at 43.2 nm. Using SEM images described in Sect “SEM images”, the size reaches 29 nm.

The zeta potential describes the surface potential on the particles in colloidal solution and the stability of the nanoparticles against agglomeration. The values of the zeta potential for samples A02, B02, and C02 are − 17.9, − 17.5 and − 16.4 mV. The negative sign means the charge on the surface of the particle is negative. This leads to repulsion force between these nanoparticles to prevent agglomeration and making stability for the particles. The higher is the zeta potential, the more stable are the nanoparticles. Several studies have reported smaller magnitude of zeta potential. For instance A. Singh et al. used Ag nanoparticles to overcome hepatocellular ailments with zeta potential of − 11.3 mV^50^.

### X-ray diffraction

**Figure 8:**
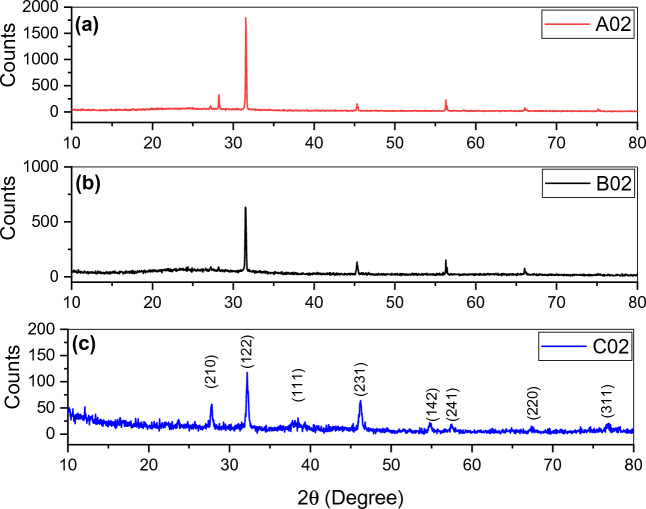
X-ray diffraction results for structural characterization of samples (**a**) A02, (**b**) B02, and (**c**) C02.

The XRD was measured in the range of diffraction angle from 5° to 80°. It presents the crystalline size and the structure of the SNPs that are characterized by the observed peaks (Fig. 8). The distinct diffraction peaks of the 2Ɵ values of 27.81°, 32.16°, 38.12°, 44.3°, 46.21°, 54.83°, 57.39°, 64.42° and 77.45° are indexed to (210), (122), (111), (200), (231), (142), (241), (220) and (311) planes correspond to a face-centered cubic structure (fcc) and are crystalline in nature^51,52^, (JCPDS file no. 84-0713 and 04-0783). There is an increase in the relative intensity of the peaks as the laser exposure time increases and there is a shift toward a lower 2Ɵ value that could be due to the change in the structure of the formed SNPs.

### FTIR spectra for SDS-AgNPs

**Figure 9:**
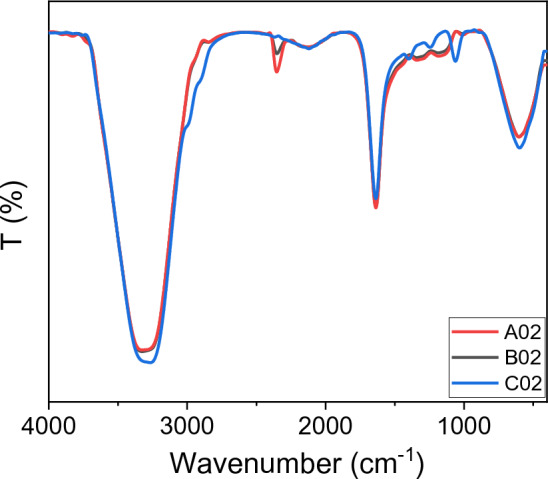
FTIR spectra for the samples A02, B02, and C02.

Infrared absorption measurements were conducted using a FTIR spectrometer in the range of 4000–400 cm^−1^. The spectra were measured in transmission mode. Figure 9 shows the FTIR spectra of the SDS capped AgNPs samples, A02, B02, and C02. The spectra depict, for sample C02, absorption peaks at 3267.0, 1637.4, 1398.2, 1245.9, 1062.7 and 597.9 cm^−1^. The peaks correspond to O–H stretching^53–56^, N–O stretching^56^, N=O stretching^53,54^, SO2 asymmetric stretching^48^, S = O stretching^55–57^, and C–H bending^53,54,58^. The peak 3267.0 is red-shifted by 61.7 cm^−1^ to be 3328.7 cm^−1^ for sample A02 and shifted by 59.9 cm^−1^ to be 3326.8 cm^−1^ for sample B02. A peak at 2352.9 cm^−1^, absent in sample C02, appears in the spectrum of the sample A02, while it has a shift for the sample B02 to appear at 2350.9 cm^−1^. This peak was attributed to C=O stretching^59^. The peak 1637.4 cm^−1^ appears at the same frequency for all samples. The peaks 1062.7, 1245.9, and 1398.2 are red-shifted by 52.1, 61.8, and 57.8 cm^−1^ for sample A02 to become 1010.58, 1184.2, and 1340.4 cm^−1^, respectively. These peaks for sample C02 are also red shifted by 53.8, 59.8, and 57.8 cm^−1^ to be 1008.85, 1186.1, and 1340.4 cm^−1^, respectively. Finally, the peak of sample C02 at 597.9 cm^−1^ is red shifted by 5.98 cm^−1^ for both samples A02 and B02 to become 603.7 cm^−1^. The shift of peaks particularly the 1063 cm^−1^ and 1246 cm^−1^ that is related to SO2 molecule indicates capping of silver NPs by SDS as the single compound of SDS has specific fingerprint peaks at 1080 cm^−1^ and 1247 cm^−1^ that are due to symmetric and asymmetric stretching vibration of SO2 molecule, respectively^55,60,61^. A redshift of the FTIR spectra is caused by extended time of laser exposure.

### The catalytic activity of SDS capped-AgNPs

The catalytic activity study of the laser-irradiated samples was performed at room temperature (25 ± 2 °C) using the reaction of MB dye with sodium borohydride in dark conditions, following amended method reported elsewhere^62–64^. That is, the laser-synthesized SDS-capped AgNPs catalytic activity was examined for the reduction of aqueous MB to Leuco–MB in the presence of excess NaBH_4_. The A02, B02, and C02 SDS-capped AgNPs’ catalytic efficiencies have been investigated and compared with noncatalytic ones. In the noncatalytic reaction, 2 mL of MB (100 ppm) was mixed with 1 mL of 0.1 M sodium borohydride.

**Figure 10:**
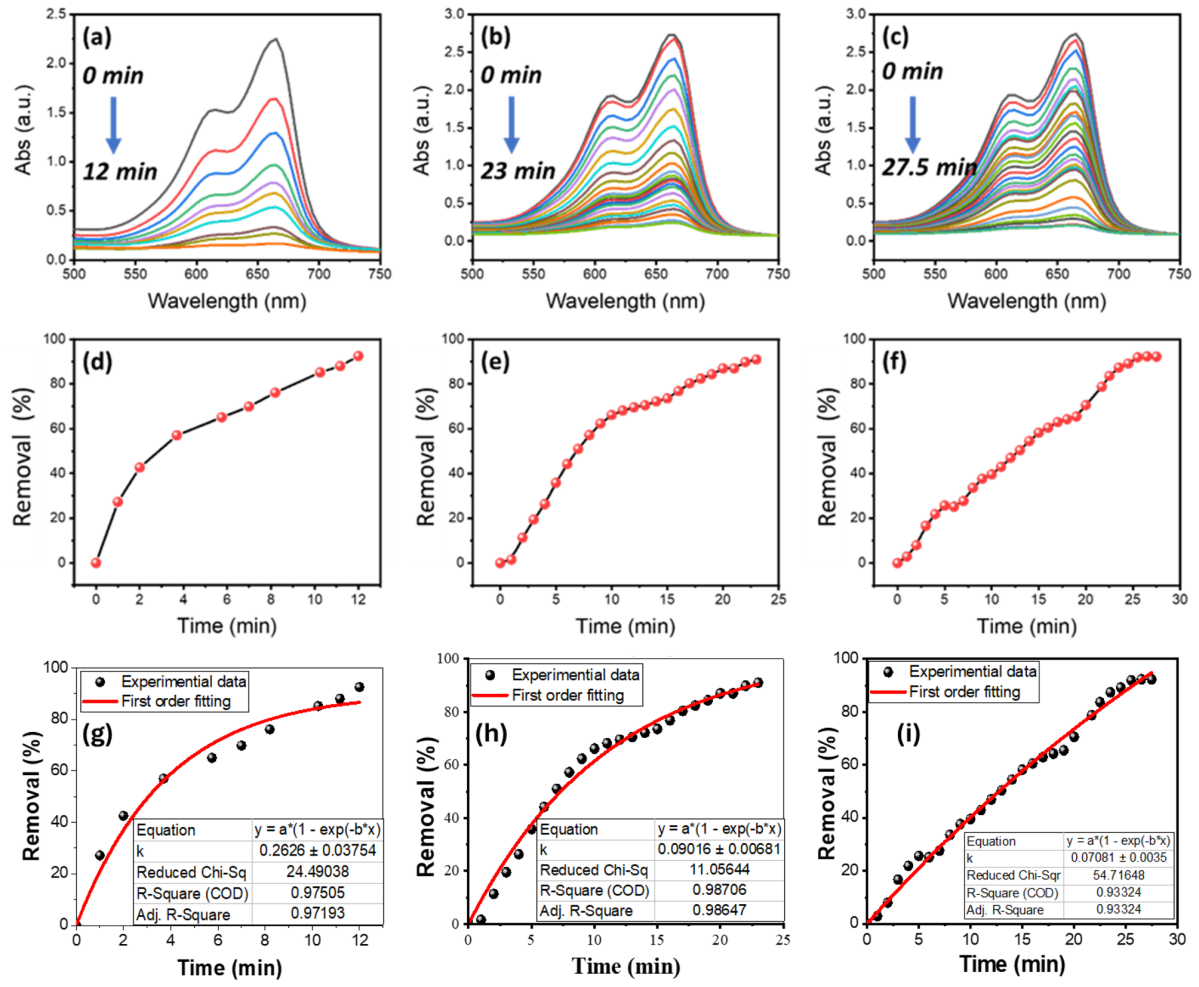
(**a**–**c**) Absorption spectra of MB in an aqueous medium at different time intervals, (**d**–**f**) catalytic activity (%); and (**g**–**i**) nonlinear first-order kinetic modeling utilizing A02, B02, and C02 catalysts respectively.

**Scheme 1:**
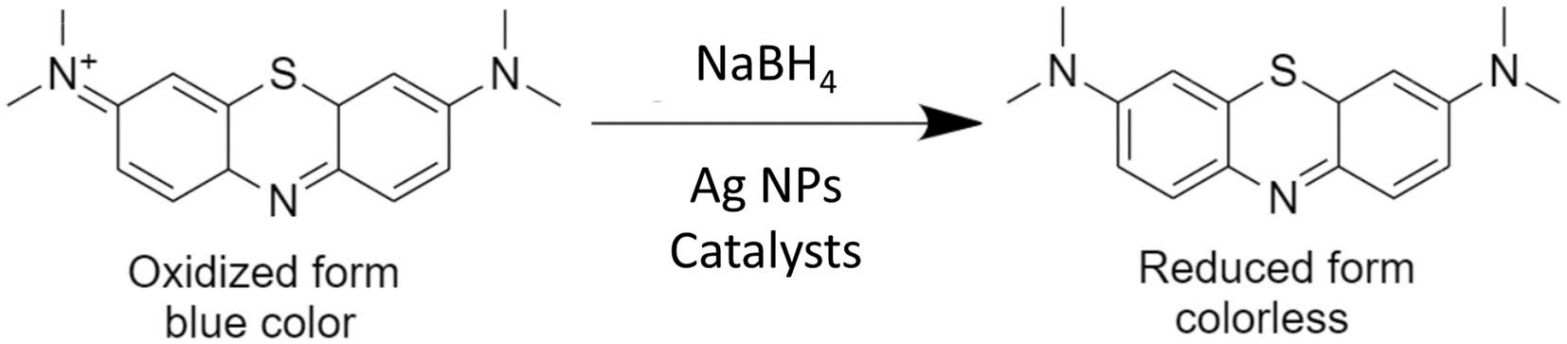
Schematic reaction of MB dye reduction using NaBH_4_ in the presence of SDS-capped Ag NPs catalyst.

In the case of the catalytic reaction using 3.5 cm^3^ cuvette, 1 mL of 0.1 M NaBH_4_ was used to examine the catalytic reduction of 2 mL of highly concentrated MB (100 ppm) by 20 μL of laser-synthesized SDS-capped AgNPs aqueous solutions from A02, B02, and C02 samples. The absorption spectra for the catalytic reactions by A02, B02, and C02 catalysts at different time intervals are shown in Fig. 10a, b, c. The absorption spectra display two peaks at 615 nm and 665 nm. The 615 nm peak is a shoulder peak for the maximum wavelength (λ_max_), while the 665 nm peak is MB’s highest absorption peak in aqueous solutions. The 615 nm peak is a high-energy band associated with a vibronic transition, and the 665 nm peak corresponds to n-π* transitions. The reduction process was found to be accelerated in the presence of our catalysts which showed a rapid reduction in the absorption intensity at 665 nm of MB solution within 12, 23, and 27.5 min, for A02, B02, and C02, respectively as shown in Fig. 10a, b, c. It was previously reported that Ag NPs generally help in the electron relay from ${{BH}_{4}}^{-}$ (donor) to MB (acceptor). ${{BH}_{4}}^{-}$ ions are nucleophilic, while MB (cationic dye) is electrophilic in nature relative to the AgNPs. Therefore, the NPs accept electrons from ${{BH}_{4}}^{-}$ ions and deliver them to the MB, as shown in Scheme 1^65^.

The MB removal percentages (Removal%) have been determined using Eq. (7) utilizing the absorption peak at 665 nm.

7 $$Removal \% = \frac{{A_{o} - A_{s} }}{{A_{o} }} \times 100$$

where ${\text{A}}_{\text{o}}$ represents the initial absorbance at time zero, and ${\text{A}}_{\text{s}}$ corresponds to the absorbance of the sample at a specific time. The results of catalytic MB removal% versus reaction time are presented in Fig. 10d, e, f for A02, B02, and C02 SDS-capped AgNPs, respectively. Also, the rate constant of the reaction has been determined from applying the nonlinear form of the first order kinetic model, Eq. (8), as shown in Fig. 10g, h, i for A02, B02, and C02 SDS-capped AgNPs, respectively.

8 $$A_{s} = A_{0} \left( {1 - e^{{ - k_{app} t}} } \right)$$

where ${\text{k}}_{\text{app}}$(min^−1^) is the rate constant for the dye removal reaction and t (min) is the reaction time.

**Figure 11:**
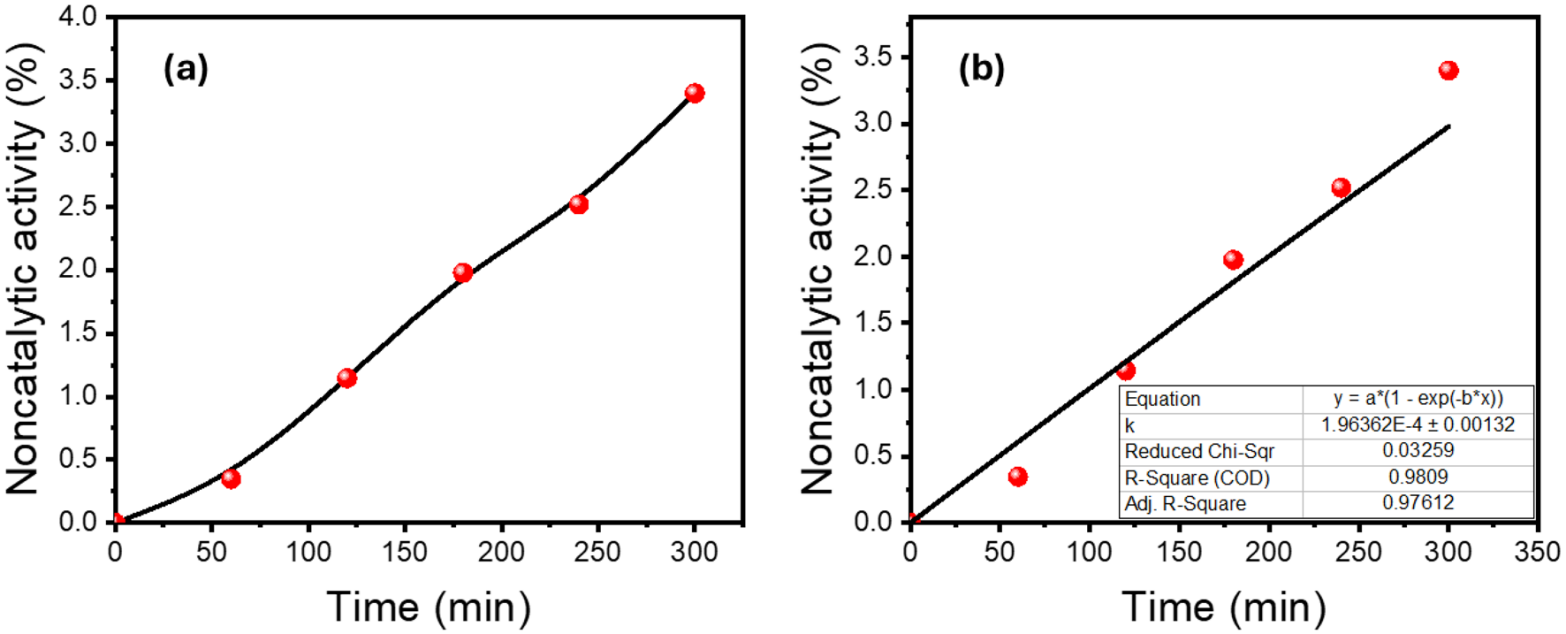
(**a**) Removal % of MB in the non-catalytic reaction and (**b**) their first order kinetic modeling with *k*_*app*_ of 0.000196 min^−1^.

Table 4 shows values of MB removal%, optimized time, K_app_ and ratios of K_apps_’ in the presence and absence of our SDS-capped AgNPs catalysts. The absorption peak at 665 nm for MB dye was found to decrease rapidly in the case of A02 NPs with an increase in the reaction time to 12 min. The removal% reached about 92.52% (Fig. 10d) with a rate constant of 0.2626 min^−1^(Fig. 10g). The spectra in Fig. 10b, c indicate that the MB dye has been degraded at a lower rate within 23 and 27.5 min, in the case of B02 and C02 catalysts, respectively. For B02 SDS-capped AgNPs, the removal% reached about 91.02% (Fig. 10e) with a rate constant of 0.09016 min^−1^ (Fig. 10h). Whereas removal% of 92.28% (Fig. 10f) and a rate constant of 0.07081 min^−1^(Fig. 10i) were reached using C02 catalyst within 27.5 min. Based on the data presented in Fig. 11a, it can be observed that the non-catalytic reaction resulted in a removal percentage of 3.7% within a time frame of 300 min. Additionally, the kinetics of this reaction can be modeled using a pseudo-first-order approach, with an apparent rate constant ${k}_{app}$’of 0.000196 ${min}^{-1}$ as depicted in Fig. 11b. It is worth noting that this rate constant indicates an exceptionally slow rate of removal for a high concentration of MB, suggesting a sluggish non-catalytic reaction.

**Table 4:** Data obtained from the catalytic MB degradation in the absence and presence of our SDS capped-AgNPs catalysts.

Catalyst	Laser exposure time (min)	MB removal (%)	Removal time (min)	*k*_*app*_ (min^−1^)	R^2^	Root-MSE (SD)	Rate of increase $=\frac{{k}_{app\, with\, catalyst}}{{k}_{app\, with\, no\, catalyst}}$
A02	15	92.52	12	0.2626	0.97505	0.03754	~ 1337.34
B02	30	91.02	23	0.09016	0.98706	0.00681	~ 459.16
C02	45	92.28	27.5	0.07081	0.93324	0.0035	~ 360.61
Nil	Nil	3.70	300	0.000196	0.9809	0.00013	Nil

The rate of increase for the catalytic removal of MB can be determined by using the equation proposed by Alfryyan et al.^63^.

9 $$rate\, of\, increase = \frac{{k_{app\, with\, catalyst} }}{{k_{app`\, with\, no\, catalyst} }}$$

The catalytic rate of increase reached ~ 1337, 459, and 361 for A02, B02, and C02, respectively, relative to the noncatalytic reaction. The findings indicate that the A02 reaction exhibited the fastest reaction rate among the tested catalytic reactions when subjected to a high concentration of the MB dye. This could be attributed to the fact that the A02 sample contains the largest quantity of the AgNPs and the smallest ratio of SDS compared to the B02 and C02 samples, as previously suggested by the absorption spectra in Fig. 1a and EDX spectra and their analysis in Fig. 6 and Table 3. Whereas the ratios of Ag/Na weight% reached 1.378, 0.776, and 0.048 for A02, B02, and C02, respectively. That is, as the laser exposure time extended to 45 min, the rate of MB dye removal in the A02 sample increased, which corresponded to a rise in the concentration of elemental silver. Also, the differences in the micro and nanomorphologies as shown in Fig. 4 may affect the surface area and hence affect the catalytic performance. The observed decrease in the B02 and C02 samples could potentially be due to the inhibitory effect of SDS, employed as a capping and stabilizing agent, on the catalytic activity of the AgNPs. This inhibition could be a consequence of the electrostatic attraction between the AgNPs and SDS. Based on our investigation into the impact of alkaline and acidic conditions on the degradation of methylene blue (MB) using the optimized SDS-capped AgNPs sample, as shown and described in the supplementary data (Fig. S1), we observed that degradation efficiency decreases under acidic conditions but improves in alkaline conditions. In other words, pH significantly influences the degradation process. Alkaline conditions enhance MB degradation, while acidic conditions hinder it. These findings align with previously reported data by Perera et al.^66^.

Our optimized SDS-capped AgNPs sample that was prepared at 45 min demonstrated a high efficiency in the catalytic degradation of methylene blue (MB) dye, achieving 92.5% dye removal in just 12 min with a rate constant of 0.2626 min^−1^. In comparison to other AgNPs-based catalysts synthesized via green routes (as indicated in Table 5), the performance of our designed catalysts stands out significantly^62,67–71^. For instance, a study uses l-histidine capped silver nanoparticles (His-AgNPs) for the degradation of MB dye, reported 98% dye removal in 40 min^72^. Another research utilized marine algae-mediated silver nanoparticles (AgNPs) for the degradation of MB, reported process completion in 20 min with a rate constant of 0.106 min^−1^^73^. In another study, AgNPs were loaded on polyvinyl alcohol sponges, which showed a rate constant of 13.7 × 10^−3^ s^−1^ for MB reduction^74^. Lastly, research that used in-situ fabricated AgNPs on TiO_2_ for 4-nitrophenol reduction reported a rate constant as high as 394 × 10^−3^ s^−1^^75^. In conclusion, our optimized SDS-capped AgNPs sample shows a promising performance in terms of both reaction time and efficiency when compared to other AgNPs-based catalysts reported in the literature. This highlights the potential of our method for industrial applications, particularly in sectors requiring efficient dye degradation.

**Table 5:** Comparative analysis of our designed catalysts and AgNPs-based catalysts synthesized via green routes.

Catalyst	Source of synthesis	C_catalyst_, V_catalyst_	C_dye_, V_dye_	C_NaBH4_, V_NaBH4_	*K*_*app*_ or time for complete reduction	Ref
GC-capped Ag NPs	Green coffee extract	500 µg/mL, 100 µL	50 ppm, 1 mL	0.1 M, 1 mL	0.2867 min^−1^, 12 min	^62^
GSH-Ag NPs	Glutathione (GSH)	7.5 × 10^−9^ mol/dm^3^, 3.9 mL	0.32 mM, 1 mL	0.1 mL, 0.05 mol. dm^−3^	0.265 min^−1^, 18 min	^67^
LC-capped Ag NPs	*Litchi chinensis* (LC) fruit extract	10 mg (powder)	10 ppm, 50 mL	Photocatalytic	0.0335 min^−1^, 50 min	^68^
Extracellular & intracellular Ag NPs	Actinomycetes strains	11.3 μg/mL, 1 mL	5 ppm, 1 mL	Photocatalytic	0.018 min^−1^, 75 min	^69^
Ag NPs	*P. quassioides* bark extract for Ag NPs synthesis	0.5 mL	1 µM, 1.5 mL	0.01 M, 1 mL	0.034 min^−1^, 15 min	^70^
GO-Ag NPs					0.038 min^−1^, 15 min	
Biogenic Ag NPs	*Piper chaba* stem extracts	53.9 mg/L, 100 µL	2 ppm, 2 mL	600 ppm, 1.5 mL	0.17 min^−1^, 12 min	^71^
A02	Laser ablated at 45 min	20 µL	100 ppm, 2 mL	0.1 M, 1 mL	0.2626 min^−1^, 12 min	Present study
B02	Laser ablated at 30 min				0.09016 min^−1^, 23 min	
C02	Laser ablated at 15 min				0.07081 min^−1^, 27 min	

## Conclusion

SDS -capped Ag nanoparticles of varying sizes were synthesized using a pulsed Nd:YAG laser at 532 nm at different exposure times. The nanoparticle sizes, confirmed by SEM images, were modeled using Lorentz fitting to the experimental absorption data. The estimated sizes were 25.8, 22.3, and 23.7 nm for the three tested samples. EDX analysis verified the presence of silver nanoparticles in accordance with the absorbance spectra for silver content. As laser exposure time increased up to 45 min, the content of silver and carbon rose, while oxygen and sodium decreased. The synthesized SDS-Capped AgNPs showed promising results in catalysis, with the sample that was prepared at 45 min achieving 92.5% MB dye removal in 12 min at a rate constant of 0.2626 min^−1^. The samples that were prepared at exposure times of 30 and 15 min demonstrated slightly lower rates, achieving around 91–92% dye removal within 23 to 27.5 min, with rate constants of 0.09016 min^−1^ and 0.07081 min^−1^, respectively. Extrapolating on the current results, we anticipate that sample exposure to laser for further prolonged time would results in complete MB removal at higher rate. These findings hold significant industrial implications, particularly for sectors requiring efficient dye degradation, such as textile and water treatment industries. The high efficiency and speed of dye removal demonstrated by the synthesized silver nanoparticles could lead to cost-effective and environmentally friendly solutions. Looking ahead, further optimization and scaling of this method could pave the way for its broader adoption in various industrial applications. Additionally, exploring the potential of these nanoparticles in other catalytic processes could be a promising direction for future research.

### Supplementary Information

Supplementary Figures.

## Data Availability

The datasets used and/or analysed during the current study available from the corresponding author on reasonable request.
