# Supplementary material for: Quick methylene blue dye elimination via SDS-Ag nanoparticles catalysts
Source: Sci Rep. 2024 Jul 2;14:15227. doi: 10.1038/s41598-024-65491-6 (PMC11220135; doi:10.1038/s41598-024-65491-6)
Supplement: Supplementary file 1 — Supplementary Figures. [file 41598_2024_65491_MOESM1_ESM.docx]

Figure S1. Effect of pH on the degradation of MB by SDS-capped AgNPs.
